# Three-Dimensional Imaging of Human Myocardial Collagen Fibers Using Trypan Blue Staining

**DOI:** 10.17691/stm2025.17.5.04

**Published:** 2025-10-31

**Authors:** A.A. Beketova, O.V. Kirik, D.E. Korzhevskii

**Affiliations:** Laboratory Assistant Researcher, Laboratory of Functional Morphology of the Central and Peripheral Nervous System, Department of General and Specific Morphology; Institute of Experimental Medicine, 12 Academician Pavlov St., Saint Petersburg, 197022, Russia; PhD, Senior Researcher, Laboratory of Functional Morphology of the Central and Peripheral Nervous System, Department of General and Specific Morphology; Institute of Experimental Medicine, 12 Academician Pavlov St., Saint Petersburg, 197022, Russia; MD, DSc, Professor of the Russian Academy of Science, Head of Laboratory of the Functional Morphology of the Central and Peripheral Nervous System, Head of the Department of General and Specific Morphology; Institute of Experimental Medicine, 12 Academician Pavlov St., Saint Petersburg, 197022, Russia

**Keywords:** trypan blue, dye fluorescence, confocal laser microscopy, collagen fibers, human myocardium

## Abstract

**The aim of the study** was to estimate the availability of trypan blue staining of human myocardium sections to investigate cardiac collagen fibers, in particular, their three-dimensional reconstructions.

**Materials and Methods:**

Pretreated with phosphomolybdic acid sections of human myocardium (n=9) were stained with 1% aqueous trypan blue solution (Panreac, Spain) (patent application RU 2024/22564). Parallel sections were stained using the similar technique with 2% aqueous solution of aniline blue (Unisource Chemicals Pvt. Ltd., India). The obtained preparations were analyzed using a light microscope Leica DM750 (Leica, Germany) and a confocal laser microscope LSM800 (Carl Zeiss AG, Germany). Threedimensional reconstruction was performed using software ZEN-2012 (Carl Zeiss AG, Germany).

**Results:**

Trypan blue appeared to stain collagen fibers selectively. Loose thick fibers were stained more intensively than thin fibers, which were not available for detailed examination with bright-field microscopy. The cytoplasm of cardiomyocytes remained unstained. Aniline blue appeared to stain thin fibers relatively more intensively, and its non-specifically stained the cytoplasm of cardiomyocytes.

Fluorescence of collagen fibers stained with trypan blue was excited with a 640 nm laser. Autofluorescence of the cytoplasm of cardiomyocytes was excited with a 488 nm laser. Three-dimensional reconstruction of endomysium showed that it was a net. Therefore, it became possible to describe the lower-order structures being the part of thick fibers. Three-dimensional reconstruction enabled to distinguish the orientation of the fibers and their cross section differentiating it from tangential one.

**Conclusion:**

The study findings showed trypan blue staining of human cardiac section to be an effective method to reveal collagen fibers, study their structure with bright-field microscopy using confocal laser microscopy and perform detailed three-dimensional reconstructions. The advantages of the method compared with other techniques of three-dimensional imaging of collagen fibers consist in the accessibility of reagents, high staining selectivity of the connective tissue matrix, the ability to obtain contrast and detailed images of the studied objects with high resolution.

## Introduction

Collagen fibers are the main components of extracellular matrix and determine a number of its properties and functions. Firstly, collagen is the source of rigidity of extracellular substance and the body in general, i.e., it provides deformation-resistant ability [1, 2]. It is achieved due to the proper mechanical properties of a collagen fiber caused by its molecular structure, as well as the spatial fiber organization. Secondly, in collagen molecules there are the sites of binding cell adhesion molecules (integrins), fibronectin and laminin, as well as glycosaminoglycans, the collection of which can regulate the activity of cells [3] including that by implementing a mechanotransduction process [1, 4]. The responses of cells to mechanical stimuli came to knowledge, primarily, due to the studies carried out on cell cultures. So, for human mesenchymal stem cell culture it was found that decreased mechanical load on tissue primarily causes differentiation into an adipogenic line, not an osteogenic one [5]. As for the culture of stem cells of striated skeletal muscles, imparting elastic properties to the substrate similar to the properties of the native extracellular matrix was stated to enable to retain the cell capability to self-renewal, which is usually lost *in vitro* [6]. Other studies revealed the reorientation of osteosarcoma cells and actin fibrils in cytoplasm in relation to the cyclic load direction, the frequency of the load and elastic properties of the matrix [7].

A response to mechanical signals extends beyond the reactions on a cellular level, and finally results in changing the matrix surrounding the cells — the matrix remodeling. The process is being under active study within the context of pathological myocardial conditions. The preloading increase in the right ventricle causes the collagen metabolism change in the heart with collagen synthesis prevailing that promotes the increase in the myocardial mass due to a connective tissue component [8]. Significant alteration of the intracardiac hemodynamics resulting from the tricuspid atresia causes the thickening of collagen fibers of different organization levels [9]. Both: the fibers surrounding vessels and cardiomyocyte bundles and the fibers forming the crossbridges between the collagen sheath of some cardiomyocytes are subjected to thickening [9]. An important direction in studying the connective tissue fibers is the search for therapeutically significant molecular targets, the action on which can prevent or decrease fibrosis [10, 11].

Currently, there are several ways of collagen imaging available for collagen matrix researchers. Firstly, there are common staining techniques. Masson staining is used to stain collagen fibers with aniline blue orthochromatically. The approach is effective to assess fibrosis areas [12]. Additionally, aniline blue can be applied as a single stain [13]. The stain is meant for bright-field microscopy; however, it is inappropriate for confocal laser microscopy, which enables to create three-dimensional reconstructions of sections. A routine method of van Gieson’s picro-fuchsin staining has a similar drawback: there are no data that the sections stained by the method are available for studying using confocal microscopy. The second method is picrosirius red staining [14], which in contrast to aniline blue, is able to fluoresce [15]. However, the work [16], where, in particular, collagen fibers were 3D-imaged using this dye, showed its affinity for connective tissue to depend drastically on fixation type. Thirdly, there is a method of the second harmonic regeneration [17]. It enables to image the position of collagen fibers in the sample volume and make calculations to construct spatial models of connective tissues [12, 18]. Although the method was developed based on multiphoton microscopes, it is available not for all optical devices of this category resulting in imposing the restrain on the technique. Fourthly, scanning electron microscopy is suggested to be used; however, threedimensional images of intercellular substance fibers require a preliminary procedure of matrix decellularization [9] that makes it impossible to study the interaction of cells with matrix.

The abovementioned disadvantages of the existing techniques cause the search for other decisions. Trypan blue staining can serve the alternative option. Until recently, trypan blue has been used to assess cell survival under different conditions due to its ability to penetrate to dead cells only [19], and in eye surgery to image the eye structures [20]. Moreover, trypan blue is used for the vital staining of the connective tissue [21]. Since it can fluoresce, trypan blue appears to be promising for staining sections aiming to further study using confocal laser microscopy.

**The aim of the study** was to assess the availability of trypan blue staining of human myocardium sections to investigate cardiac collagen fibers, in particular, their three-dimensional reconstructions.

## Materials and Methods

The study was focused on human myocardial sections (n=9). The blocks of fixed in formalin and embedded in paraffin samples were received from the histological archive of the Department of General and Specific Morphology, Institute of Experimental Medicine (the archive was established based on local Ethics Committee decision, verdict No.58-9/1-684, December 11, 2009). There were prepared the sections 5 μm thick (n=18). Prior to staining, paraffin was removed from the sections using xylene followed by rehydrating the sections in ethanol in descending concentration.

Trypan blue staining (Panreac, Spain) was performed according to the following technique (patent application RU 2024/22564): 1% aqueous solution of phosphomolybdic acid was applied on the sections for 15 min at room temperature, which was removed afterwards. Then, without washing, the preparations were applied with 1% aqueous solution of trypan blue for 5 min under the same conditions. After that the dye was removed from the sections, and the sections were washed with water. The staining with 2% aqueous solution of aniline blue (Unisource Chemicals Pvt. Ltd., India) was performed according to the similar technique. After staining all sections were dehydrated in isopropanol and xylene, and placed into the permanent medium Bio Mount HM (Bio-Optica, Italy).

The preparations were imaged using a light microscope Leica DM750 and a digital camera ICC50 (Leica Microsystems, Germany; lens Plan 10×/0.22), along with a confocal microscope LSM800 (Carl Zeiss AG, Germany; lens Plan-Apochromat 63×/1.40 Oil DIC M27). Three-dimensional reconstruction was carried out using the software ZEN-2012 (Carl Zeiss AG, Germany). The fiber thickness was measured in the program ImageJ using the built-in tool “straight” and “measure”. The findings were statistically processed using Microsoft Excel (Microsoft, USA).

## Results

**Figure 1.**
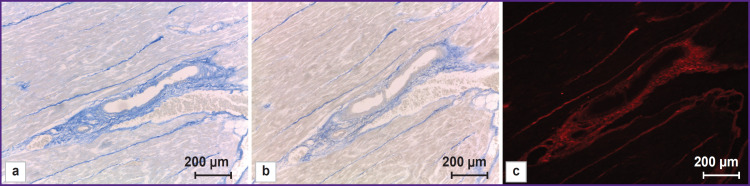
Connective tissue of human myocardium: (а) aniline blue staining, parallel to (b) and (c) section; (b), (c) trypan blue staining, one and the same section; (а), (b) bright- field microscopy; (c) confocal laser microscopy; 10×

Trypan blue staining gave a picture different in contrast in bright-field microscopy and confocal microscopy (Figure 1 (b), (c)). Visually, trypan blue highly selectively stained the connective tissue fibers. Visible staining intensity depended on the preparation time of the phosphomolybdic acid and a dye. The best staining result was achieved when using the acid and the dye prepared immediately prior to the staining. A dye prepared 3 months before had the decreased affinity for the connective tissue that was manifested by the pale staining of the connective tissue. Phosphomolybdic acid in an aqueous solution even if kept in an opaque package failed to provide high staining selectivity in as little as a week.

In bright-field microscopy the fibers of different thickness were imaged differently. Large collagen fibers, 7.583±3.426 μm thick on average, were intensely stained. Thin, detached fibers, 1.589± 0.421 μm thick on average, were poorly stained, while the fibers 0.557±0.174 μm in diameter on average, were hardly stained. The lowest fiber diameter revealed was 0.279 μm. The connective tissue interlayers surrounding each cardiomyocyte (endomysium) were also stained, although it was impossible to see and much less reveal the fibers constituting the interlayers in case of low power of the lens. Aniline blue appears to stain this fiber type relatively more intensely (Figure 1 (а)). However, it should be noted that aniline blue has some, although minor, affinity for cardiomyocyte cytoplasm changing it into grey color that is not typical for trypan blue.

**Figure 2.**
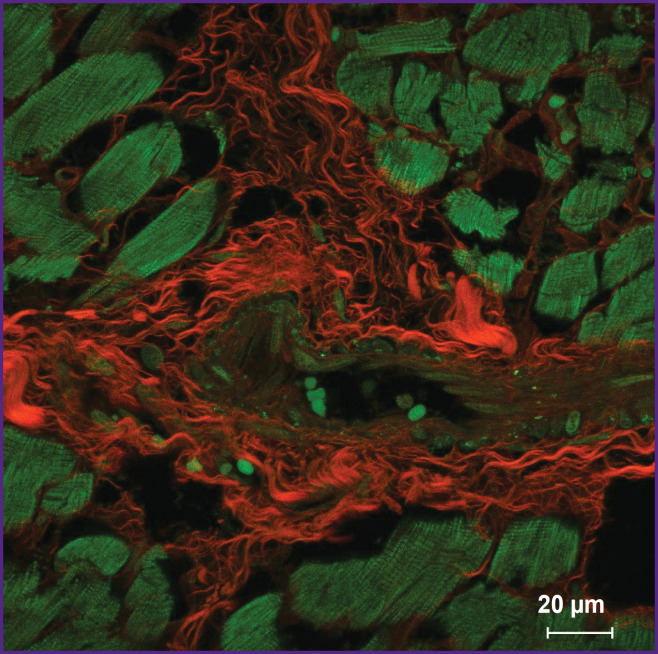
Human myocardium in fluorescent staining with trypan blue Orthogonal projection of 18 optical slices, 0.33 μm thick. Red — fluorescence of trypan blue bound to collagen fibers; green — autofluorescence of the cytoplasm of cardiomyocytes, erythrocytes and the nuclei of smooth muscular cells of vessel muscular layer; 63×

**Figure 3.**
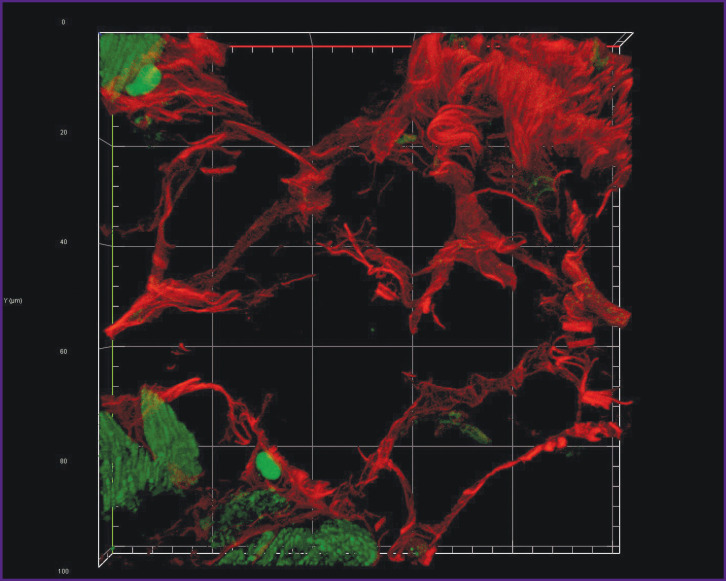
Three-dimensional reconstruction of the connective tissue fibers of human myocardium when stained with trypan blue in the space by 19 non-overlapping optical sections Dimensions along Х axis — 100 μm, along Y axis — 100 μm, along Z axis — 6 μm. Red — fluorescence of trypan blue bound to collagen fibers; green — autofluorescence of cardiomyocyte cytoplasm; 63×

The fluorescence of collagen fibers stained with trypan blue can be observed when excited by laser wavelength 640 nm (Figure 2). The cytoplasm of nonstained cardiomyocytes fluoresced when excited by laser wavelength 488 nm. An initial image had high contrasting effect; therefore, it required no fine tuning in image processing that enabled to accelerate and simplify the image analysis process using the software (ImageJ). Moreover, compared with the images obtained using bright-field microscopy, in the images obtained using confocal microscopy the program appeared to more selectively and more precisely determine the collagen fibers including the thinnest fibers, which failed to be contrastively revealed using light optical microscopy. The fluorescent signal intensity from the fibers over 0.4 μm in diameter was comparable with the signal intensity from the fibers 4.0±1.0 μm in diameter. The minimum fiber diameter found was 0.201 μm. Three-dimensional imaging of endomysium (Figure 3) showed it to be the net, the elements of which cannot be differentiated as separate structures. It suggests the dimensions of the elements to be less than the resolution of a confocal microscope. For thick fibers it was possible to describe the low-order structures being the part of their composition (see Figure 2). The fibers, 0.315±0.040 μm thick on average, were found to be situated between the neighboring fibers over 1.5 μm in diameter passing transversely and obliquely in relation to the thicker fibers. In addition to the course of collagen fibers, three-dimensional imaging enabled to determine their cross-section: it was irregularly shaped, nearly oval. Considering a complete fiber attitude in space, it became evident that it was a cross-section not a tangential section, which had this form, that could be supposed based on one optical section.

## Discussion

Trypan blue is a generally available reagent used in medical biological studies. Its primary application is in experiments, when it is needed to assess cell viability: both — in culture [22, 23] and also in a multicellular organism at various stages of a modeled pathological process [24]. Secondly, trypan blue is used in eye surgery to visualize physiological and pathological structures, namely, an internal glial limiting layer and an epiretinal membrane [25], the anterior capsule of lens [26], as well as to reveal retinal tear [27] and contrast the trabecular meshwork in micro-invasive glaucoma surgery [28].

The abovementioned cytological techniques, as well as the vital staining of the connective tissue with trypan blue [21, 29] use the dye ability to stain tissues both: orthochromatically, and also using fluorescence. Moreover, the second property unlocks new capabilities for a wide range of fluorescence microscopy including fluorescent microscopy itself, confocal laser microscopy, high-resolution laser microscopy, multiphoton microscopy and ultrahighresolution microscopy. All mentioned methods enable to obtain detailed high-resolution images, and current processing programs — to perform three-dimensional reconstruction of sections.

Phosphomolybdic acid application made it possible to obtain selectively stained connective tissue fibers. The technique is used in Masson [30], Mallory [31], Heidenhain [32], Slinchenko [33] trichromes, as well as in picrosirius red staining [15]. Like aniline blue, trypan blue is referred to anion dyes, which have basic groups, amino groups, in particular [34]. In its turn, phosphomolybdic acid is a polyvalent anion, and binds to positively charged structures preventing the direct action of anion dyes. Phosphomolybdic acid binding to different structures is not the same: most intensively it binds to cell cytoplasm, and less intensively — to the connective tissue fibers leaving a positively changed histidine molecule available for binding to trypan blue molecule [35]. Thus, tissue pretreatment with phosphomolybdic acid provides the dye binding to collagen fibers only.

Fluorescence findings compared: in trypan blue staining according to the technique we suggested and picrosirius red staining represented in literature [15, 36] — showed trypan blue to enable to obtain high- contrast images comparable in picrosirius red staining with the pretreatment with phosphomolybdic acid. However, there is no difference in neither the dimensions of minimum-sized fibers revealed, nor high contrast of the revealed structures relative to the background. In case of trypan blue staining, the fluorescence of collagen fibers is characterized by a wavelength different from that of the fluorescence of cardiomyocyte cytoplasm; it enables to receive signals singularly. Owing to this, such staining can be used both: for selective imaging of collagen fibers, and also for co-imaging of cells and the connective tissue matrix surrounding them, moreover, without the fiber structure detailing impairment.

The three-dimensional reconstruction of the picrosirius red stained sections was performed in the study by Kim et al. [16], where the authors gave a complete protocol of producing the mouse kidney preparation starting from fixation. The illustrations published by the authors give evidence of insufficient specificity of the dye binding to the elements of kidney extracellular matrix that is most notably when compared with the findings of immunohistochemical reaction to type I collagen using the same material. In trypan blue staining, fluorescence is detected only from the connective tissue fibers, their morphological characteristics corresponding to collagen fibers [37].

The three-dimensional imaging of collagen fibers using the second harmonic generation is an effective method to study collagen. Its advantages consist in the following: it enables, firstly, to study both — native and fixed and stained tissues; secondly, obtain highresolution images [38]. The method to a greater extend is applicable to type I collagen, which has the highly- ordered structure and is able to generate the second harmonic. The second harmonic signal from type III, IV, and V collagens can be lower in intensity or missing. In this case the content of these collagens can be judged either by the total signal from a sample, or after staining, e.g., picrosirius red staining [17, 39]. Trypan blue, in its turn, binds to both: the connective tissue collagen fibers represented primarily by type I collagen, and also the basal membranes of the cells represented by type IV collagen. It should be noted that the second harmonic generation microscopy has high informativity, however, its implementation requires expensive and high-precision equipment, the installation of which is an intricate technical problem. It is likely to reduce the method availability [40].

### Study restrictions

Despite the mentioned advantages of the suggested technique, attention should be paid to the evidence base restrictions related to no parallel presentation of the findings using supplementary reference methods, such as multiphoton microscopy in the second harmonic generation mode, and immunohistochemical study for type I and III collagens.

## Conclusion

The study findings showed trypan blue staining enables to study the structure of human cardiac collagen fibers on a light optical level using fluorescent microscopy and perform detailed three-dimension reconstructions of the fibers. The suggested method has a number of advantages compared with other techniques of three-dimension imaging of collagen fibers, namely: the accessibility of reagents, high staining selectivity, the ability to obtain contrast and detailed images of the studied objects with high resolution.
